# Comparative Investigations of AlCrN Coatings Formed by Cathodic Arc Evaporation under Different Nitrogen Pressure or Arc Current

**DOI:** 10.3390/ma14020304

**Published:** 2021-01-08

**Authors:** Adam Gilewicz, Tatyana Kuznetsova, Sergei Aizikovich, Vasilina Lapitskaya, Anastasiya Khabarava, Andrey Nikolaev, Bogdan Warcholinski

**Affiliations:** 1 Faculty of Mechanical Engineering, Koszalin University of Technology, 2, Śniadeckich, 75-453 Koszalin, Poland; adam.gilewicz@tu.koszalin.pl (A.G.); bogdan.warcholinski@tu.koszalin.pl (B.W.); 2 Nanoprocesses and Technology Laboratory, A.V. Luikov Institute of Heat and Mass Transfer of National Academy of Science of Belarus, 15, P. Brovki str., 220072 Minsk, Belarus; kuzn06@mail.ru (T.K.); vasilinka.92@mail.ru (V.L.); av.khabarova@mail.ru (A.K.); 3 Research and Education Center “Materials”, Don State Technical University, 1, Gagarin sq., 344003 Rostov-on-Don, Russia; andreynicolaev@eurosites.ru

**Keywords:** AlCrN, arc current, structure, hardness, adhesion, wear

## Abstract

Tools and machine surfaces are subjected to various types of damage caused by many different factors. Due to this, the protecting coatings characterized by the best properties for a given treatment or environment are used. AlCrN coatings with different compositions, synthesized by different methods, are often of interest to scientists. The aim of the presented work was the deposition and investigation of two sets of coatings: (1) formed in nitrogen pressure from 0.8 Pa to 5 Pa and (2) formed at arc current from 50 A to 100 A. We study relationships between the above technological parameters and discuss their properties. Coatings formed at nitrogen pressure (p_N2_) up to 3 Pa crystallize both in hexagonal AlN structure and the cubic CrN structure. For p_N2_ > 3 Pa, they crystallize in the CrN cubic structure. Crystallite size increases with nitrogen pressure. The coatings formed at different arc currents have a cubic CrN structure and the crystallite size is independent of the current. The adhesion of the coatings is very good, independent of nitrogen pressure and arc current.

## 1. Introduction

During the last few decades, extensive works have been carried out on the production and testing of the properties of thin coatings which can be applied in industry, medicine, electronics as protective and decorative coatings, memory technology, etc. [1,2].

Currently, it is recommended to eliminate cooling and lubricating liquids from technological processes due to economic factors and ecological requirements. Hard coated tools are used in the vast majority of metal machining operations. The most commonly used coatings are based on titanium nitride and chromium nitride [1,3]. CrN, however, is characterized by a better resistance to high temperatures compared to TiN [3]. Such coatings improve the wear resistance of cutting tools [4,5]. They have high hardness [6], a relatively low coefficient of friction [7,8], good adhesion to the substrate [9,10], chemical stability, and resistance to corrosion [11]. It was also shown that coatings may reduce the heat generated in the cutting process due to friction, and thus increase the durability of tool blades [12]. The relatively low resistance to oxidation of two-component coatings, e.g., CrN, means that they are not able to meet the expectations of the most demanding customers.

The improvement of the thin coating properties was achieved by doping with elements, such as Si, B, C, O, Al, V, and others [13,14,15,16,17]. The addition of Si enables to enhance the hardness and Young’s modulus of the Cr–N system, but Si has a negative influence on the tribological properties of the coatings—the friction coefficient increases and wear resistance decreases. This effect depends on the silicon concentration in the coating [13]. CrCN shows denser structures and significantly increased hardness and adhesion strength. It is characterized by improved corrosion resistance, the lowest corrosion current density, and the highest corrosion potential [14]. The addition of boron to CrN results in notable refinement in grains. As a result of the fine-grained structure strengthening and the solution strengthening caused by boron, CrBN coating shows improved hardness compared to CrN but relatively low adhesion [15]. Vanadium addition improve the hardness and toughness of CrN film. The coefficient of friction is also reduced for CrVN coatings [16]. Oxygen can decrease of the mean crystallite size, increase the hardness of the coating up to 28 GPa, and improve the thermal stability [17]. One of the most promising coatings seems to be AlCrN due to its very good resistance to oxidation, high hardness at elevated temperature, and good tribological properties [18]. This is because AlCrN can oxidize and form on the surface aluminum oxide Al_2_O_3_, blocking oxygen diffusion into the coating. Additionally, α-phase Al_2_O_3_ of aluminum oxide provides good tribological properties [19]. 

Investigations assessing the importance of the chemical composition of the coating to its structure and mechanical properties revealed the transformation of the metastable Al_x_Cr_1−x_N solid solution with a cubic structure (fcc) type B1 into a hexagonal structure B4, occurring at the Al/(Al + Cr) ratio from 0.6 to 0.75 (depending on the deposition method) [20]. The transformation of c-AlN → h-AlN occurs spontaneously and irreversibly. The coatings containing the AlN phase with a cubic structure (c-AlN) are characterized by better mechanical properties than the hexagonal AlN (h-AlN) phase [20,21]. Therefore, in order to obtain the coating with fcc structure and good mechanical properties, i.e., high hardness and good resistance to wear, the aluminum concentration in the coating ought to be attended to.

AlCrN coatings were formed from individual Al and Cr [22,23,24] cathodes and AlCr alloy cathodes with different fixed composition: Al_50_Cr_50_ [25], Al_60_Cr_40_ [26], Al_70_Cr_30_ [27,28], Al_80_Cr_20_ [29], and other [17,25,27]. They can be formed by many methods, including magnetron sputtering [19,20], high-power impulse magnetron sputtering (HIPIMS) [1,22,25], or cathodic arc evaporation [20,24,27,28,30]. The high degree of plasma ionization, high deposition rate, high density and good adhesion of the coatings to the steel substrate are the advantages of the latter method. Its disadvantage is a large number of defects (macroparticles, craters) on the coating surface, ranging in size from nanometers to single micrometers [30]. Cathodic arc evaporation systems are widely used as efficient devices in coating technology.

Coating deposition parameters such as nitrogen pressure [25,30], substrate bias voltage [24] and arc current in cathodic arc evaporation [31], as well as AlCr cathode composition [20], determine the coating properties. Although AlCrN coatings with different chemical composition are the subject of many investigations, they mainly include structure and phase composition [20,22,24,30], thermal resistance [20], and some mechanical [22,24,30] and tribological properties [22,24,27]. It has been proven that the composition of coatings [20,23] strongly influences their mechanical properties and microstructure. The technological parameter of deposition, substrate bias voltage [24,27,28], has a similar effect on the properties of the coatings. Along with the increase of Al concentration in the coating to approx. 70%, its hardness increases [20]. The coatings with a higher concentration of aluminum are characterized by lower hardness. There are few works describing the influence of nitrogen pressure on the properties of the coatings [25,30,32]. They indicate an increase in hardness and Young’s modulus with increasing nitrogen pressure during coating formation [25,30], regardless of the coating formation method, as well as changes in their phase structure. With increasing pressure, the formation of the hexagonal Cr_2_N phase [30] is possible as a result of a greater number of collisions of particles emitted from the cathode with gas particles. However, no phase transformation is also observed in the coatings formed at different nitrogen pressure, and only a change in texture [25]. A similar lack of changes in the dominant phase is observed in the coatings formed at different arc currents [31]. Until now, few authors have also presented systematic and detailed tests of coating adhesion to the substrate [23,30]. As adhesion is one of the basic factors determining the functional properties of coatings, it seems advisable to take up this topic, i.e., to assess the effect of nitrogen pressure during deposition on the adhesion of the coatings. 

Another technological parameter that can effectively improve the surface quality of coatings formed by the cathodic arc evaporation is the arc current [31]. The arc current can reduce the macroparticle amount, but the arc current change results in the plasma density change. This can alter the properties of the coatings. This effect has not been systematically tested for AlCrN coatings, and the presented results are inconclusive [31] in relation to our preliminary research. Lin et al. found that, with increasing arc current, the grain size in the coating and its hardness decreased. According to the Hall–Petch relationship, the opposite effect should be observed.

The goal of this study was to check the influence of nitrogen pressure and arc current during deposition of AlCrN coatings from Al_70_Cr_30_ cathode on their structure, morphology, and mechanical properties including adhesion.

## 2. Experimental

### 2.1. Deposition of the Coating

To synthesize AlCrN coatings the cathodic arc evaporation method was applied. Mechanically finished substrates (Ra about 0.02 mm) were cleaned in an ultrasonic bath. After drying, they were mounted on the rotating holder in vacuum chamber. The arc sources were equipped with Al_70_Cr_30_ cathodes and were located approximately 18 cm from the substrates. The working chamber was evacuated to the pressure of 10^−3^ Pa. The substrate temperature during ion etching and coating deposition was about 350 °C. Ion etching was performed with the following parameters: argon pressure of 0.5 Pa, voltage of −600 V, time of 10 min. The first stage of forming the coating consisted of creating an adhesive layer (gradient CrN_x_) improving the coating adhesion. This process was carried out using the Cr cathode and the following parameters: arc current 80 A, nitrogen pressure increase rate—about 0.1 Pa/min to reach a pressure of 1.8 Pa. In the second stage, two sets of AlCrN coatings were deposited under following parameters:

(a) Substrate bias voltage −100 V, arc current 80 A, and nitrogen pressure (p_N2_) in the range from 0.8 Pa to 5 Pa.
(b) Substrate bias voltage −100 V, nitrogen pressure of 4 Pa, and arc current (Ic) in the range from 50 A to 100 A.

The flow of both gases used (argon and nitrogen) and gas pressure were controlled, respectively, using an MKS flow controller (MKS Instruments, Inc., Austin, TX, USA) and by a Baratron type capacity gauge (MKS Instruments, Inc., Austin, TX, USA).

Taking into account the nitrogen pressure (X) during coating formation, they were marked as AlCrN(X). This means that, for example, an AlCrN(1.8) coating was formed at p_N2_ = 1.8 Pa. The coatings formed at various arc current (Y) are marked as AlCr(Y)N. Thus, the coating designated AlCr(60)N was formed at an arc current of 60 A. 

### 2.2. Coating Investigations

The coating thickness was investigated using spherical abrasion test. Surface morphology and microstructure were studied using scanning electron microscopy (JEOL JSM-5500LV, JEOL Ltd., Tokyo, Japan). The Hommel Tester T8000 contact profilometer (Hommelwerke GmbH, Schwenningen, Germany) was used to measure the surface roughness of the coatings. For each sample, the test was conducted five times. 

The chemical composition of the coatings was determined by energy dispersive x-ray spectroscopy (EDX, Oxford Link ISIS 300, Link Analytical/Oxford Instruments, High Wycombe, UK) and wavelength-dispersive X-ray spectroscopy (WDX, ThermoScientific’s Magnaray system, Thermo Fisher Scientific, Waltham, MA, USA) methods. The crystalline structure of the coatings was investigated using grazing incidence X-ray diffraction (Empyrean PANalytical, Malvern Panalytical Ltd., Malvern, UK) with Cu-Kα radiation (0.154056 nm) and the grazing incidence geometry at 3°. For data processing, HighScore Plus with ICDD PDF 4+ Database software (The Powder Diffraction File) was applied. Diffraction images made it possible to calculate the crystallite size in the coatings using the Scherrer equation [33]. Due to the instrumental broadening of the diffraction line, the Warren and Biscoe correction method was taken into account [33].

The semi-automatic Fischerscope HM2000 hardness tester (Fischer Technology Inc., Windsor, CT, USA) equipped in WIN-HCU^®^ software (3.0, Windsor, CT, USA) made it possible to determine the hardness (H) and Young’s modulus (E). The device was equipped with a Bercovich intender three-sided pyramid, with a tip radius of 150 nm and a total included angle of 142.3°. Taking into account the coating thickness, the measurements were conducted for a fixed penetration depth of 0.3 µm, which is less than 10% of the thickness of the coating. This indentation depth avoids a change in the measured hardness due to relatively soft substrates. The coatings with high surface roughness make it impossible correct hardness measurement. Therefore, prior to the measurements, the coatings were treated using the procedure described in Ref. [12]. After polishing the coatings with fine-grained (1 μm) aluminum oxide powder to a roughness of 0.04–0.05 μm, a much smaller dispersion of the results was obtained. The average value of hardness and Young’s modulus was calculated from at least 20 measurements from all tested surface.

Coating adhesion was determined using two methods:

(a) The scratch method (Revetest^®^ by CSEM Instruments, Peseux, Switzerland) with Rockwell C type diamond indenter with a tip radius of 200 µm. At least three scratches, each of a length of 10 mm and a distance at least 3 mm from each other, were done by moving the indenter at a speed of 10 mm/min, and simultaneously increasing the load linearly from 0 to 100 N. Two characteristic damages were observed and marked: Lc_1_ for first cracks of the coatings appeared and Lc_2_ for total delamination of the coating,
(b) Daimler–Benz test. In this method, it was assumed that the assessment of coating adhesion is determined on the basis of the form and intensity of damage resulting from the indentation [34]. In this test the Rockwell indenter is pressed into the sample (coating) with the normal load of 1471 N. It is a comparative method (six-grade scale of adhesion quality) in which it was assumed that damage in the coating corresponding to the HF1-HF4 patterns (cracks and small chips) proves the proper adhesion of the coating. The occurrence of coating defects in the area surrounding the indentation, corresponding to the HF5-HF6 patterns, is evidence of insufficient adhesion of the coating.

The studies of the friction and wear of the coatings were conducted in the ball-on-disc system under the normal load L = 20 N and a sliding speed of about 0.2 m/s for about 29000 cycles (distance s = 2000 m) in dry friction conditions. As a counterpart the mirror finished Al_2_O_3_ ball with a diameter of 10 mm was applied. The measurements were conducted in moisture of about 40% at ambient temperature. The known equation kv = Vs^−1^L^−1^ was applied to calculate the wear rate (kv). In this equation V means the volume of the removed coating material. Five randomly selected cross-sectional wear profiles were applied to calculate the wear rate.

The surface investigations of the coatings were carried out using Dimension FastScan atomic-force microscope (Bruker, Santa Barbara, CA, USA) in the PeakForce Tapping QNM (Quantitative Nanoscale Mechanical Mapping) mode. The standard silicon cantilevers of NSC11 (Micromasch, Tallinn, Estonia) type with the stiffness k = 4.11 N/m and tip radius R = 10 nm were used [35].

Hysitron 750 Ubi nanoindenter (Bruker, Minneapolis, MN, USA) was applied to construct microhardness (H) and elasticity modulus (E) maps. A diamond Berkovich indenter with a tip radius of 200 nm was applied. A fused quartz calibration sample was used to calibrate the tip radius. The E and H maps with the size of 20 × 20 µm^2^ were obtained from 400 indentations at a load of 2 mN on the surface. Before measurements, the coatings surface was polished to reduce the roughness. 

## 3. Results

**Figure 1:**
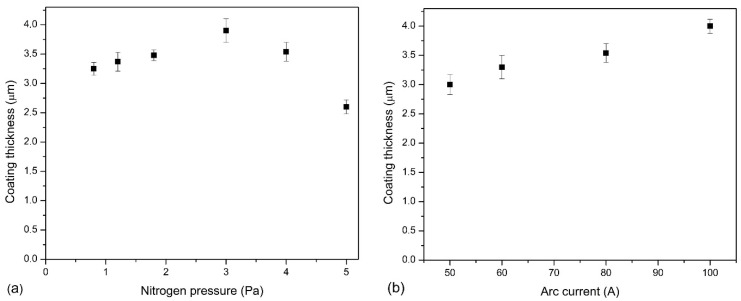
Thickness of AlCrN coatings formed at: (**a**) various nitrogen pressure, (**b**) various arc current.

As already mentioned, the coating deposition time is the same for all coatings and amounts to 120 min. Nitrogen pressure definitely affects the thickness of the resulting coatings, as shown in Figure 1a. The coating thickness ranges from 2.60 µm (p_N2_ = 5.0 Pa) to 3.9 µm (p_N2_ = 3.0 Pa). Coatings synthesized at higher p_N2_ = 4 Pa and 5 Pa present decreasing thickness. In the case of another parameter of the coating technology, the arc current, a clear tendency of the coating thickness increase with increasing current can be observed. The smallest is for coatings deposited at Ic = 50 A and is about 3 µm. As the arc current increases, the thickness increases almost linearly to a value of about 4 µm (Ic = 100 A), Figure 1b.

A similar change in coating thickness was observed by Wang et al. [30]. In the Cr-Al-N coatings formed under nitrogen pressure from 1 Pa to 2 Pa, by the multi-arc ion plating method, the maximum thickness was for the coating formed at a pressure of 1.5 Pa and then its reduction. It was attributed to the resputtering effect. In the case of coatings formed at various arc currents, the observed changes in thickness are also consistent with the literature, as an increase in arc current results in an increase in the thickness by about 30% [31], which is similar to the results shown in the presented work.

### 3.1. Chemical and Phase Composition of AlCrN Coatings

**Figure 2:**
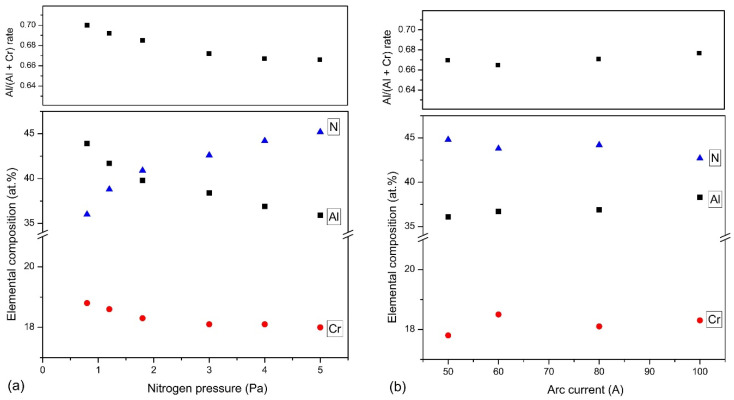
Elemental composition and Al/(Al + Cr) ratio of the coatings deposited at: (**a**) various nitrogen pressure, (**b**) various arc current.

In Figure 2, the results of the investigations on the elemental composition of the tested coatings are shown. They indicate the presence of chromium, aluminum, and nitrogen in the coatings. The tests also confirmed the presence of oxygen in all coatings in the amount of up to about 1.2% at. The nitrogen concentration in the coatings increases with the nitrogen pressure during their formation. Simultaneously, a noticeable reduction in the metallic elements chromium and aluminum is observed. The reduction in aluminum concentration is higher. This is confirmed by the calculated Al/(Al+Cr) rate in the coatings. For coatings synthesized at p_N2_ = 0.8 Pa is about 0.70, as for pure cathode and drops to about 0.666 for coatings synthesized at p_N2_ = 5 Pa, Figure 2a. This effect is probably related to lower atomic mass of aluminum. It is characterized by lower vapor density because in collisions with nitrogen it is more dispersed [36].

In AlCrN coatings formed by HIPIMS from Al_50_Cr_50_ cathodes with an increase in the N_2_/Ar ratio from 20% to 140%, an increase in nitrogen concentration in the coating from about 26% to about 51% was observed. An approximately 30% reduction in the concentration of metallic elements aluminum and chromium is also visible [25]. A similar effect is observed in the case of the coatings formed by the multi-arc ion plating from the Al_67_Cr_33_ cathode [30].

The coatings deposited at p_N2_ = 4 Pa and various arc currents are characterized by a similar chemical composition, Figure 2b. Only the coating formed at Ic = 50 A is characterized by a slightly higher, by about 2 at.%, nitrogen concentration compared to the coating formed at Ic = 100 A. In all coatings, the Al/(Al+Cr) rate is similar and amounts to 0.66–0.67.

**Figure 3:**
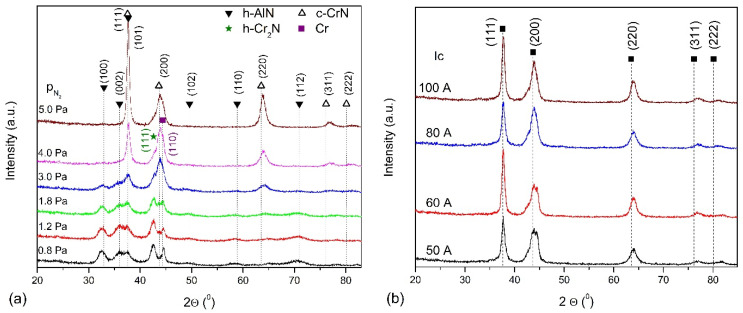
Diffraction patterns of AlCrN coatings formed at: (**a**) various nitrogen pressure, (**b**) various arc current.

Structure characterization clearly indicates that the AlCrN coatings are polycrystalline with structure dependent on nitrogen pressure during deposition. In Figure 3a, the grazing incidence XRD patterns of AlCrN coatings formed at nitrogen pressure ranging from 0.8 Pa to 5 Pa is shown. In the coating formed at the pressure of 0.8 Pa, the occurrence of h-AlN phase diffraction lines (ref. code 01-070-0354), for the angle 2Θ of approximately 32.9° (100), 36.0° (002), 37.7° (111), 49.6° (102), 58.8° (110) and 70.9° (112). Additionally, diffraction line from the hexagonal Cr_2_N phase (ref. code 04-014-1025) for an angle of 2Θ of about 42.5° (111) is visible. It can also be assumed that the line Cr_2_N (110) about 37.3° overlaps with the line (111) h-AlN. A distinct line of approximately 44.6° can be attributed to chromium (ref. code 01-077-7589). A decrease in the intensity of lines originating from the hexagonal phases of AlN and Cr_2_N, and from chromium is observed in coatings desposited at higher nitrogen pressure. At the same time, the intensity of the diffraction lines, which can be attributed to the cubic phase of chromium nitride (ref.code 04-004-6868), increases. These are lines positioned at about 37.6°—(111) plane, 43.8°—(200) plane, 63.5°—(220) plane, 76.1°—(311) plane and 80.1°—(222) plane. In the coatings formed under nitrogen pressure in the range from 1.8 Pa to 4 Pa, diffraction lines from three phases, hexagonal AlN and Cr_2_N and cubic CrN are observed. The lines from the hexagonal phase disappear almost completely in the coatings synthesized at p_N2_ = 4 Pa. Formation of the AlN cubic phase cannot be excluded, however, its identification is difficult. These phases are characterized by the same Fm-3m space group (225) due to the fact that the diffraction line positions in the cubic AlN and CrN phases differs only slightly.

For the lowest N_2_/Ar ratio, the resulting structure is rather amorphous with hardness of around 11–13 GPa and a high wear rate of around 10^−5^ mm^3^/Nm. An increase in this ratio in the process of AlCrN coating forming by HIPIMS, diffraction lines are observed only for cubic CrN phase. The intensity of the (111) CrN line with an increase in N_2_/Ar decreases as well as the (220) CrN line, while the intensity of the (200) CrN line increases [25]. For coatings formed by the mult-arc ion plating method, with increasing nitrogen pressure, the intensities of lines (111) and (200) decrease, but the line (111) Cr_2_N appears [30]. 

The coatings formed at different arc currents have a similar diffraction patterns, Figure 3b. The diffraction lines correspond to the major lines of the cubic CrN phase. According to the above standard, the ratio of the intensities of the most intense (200)/(111) diffraction lines is 1.22, while for coatings formed at different arc currents ranges from 0.61 to 0.88. This shows that these coatings are strongly textured.

The structure of AlCrN coatings formed at different arc currents is fcc-CrN phase. The increase in the arc current does not cause significant changes in the intensity of the observed diffraction lines, while their widening may result in grain refinement [31].

**Figure 4:**
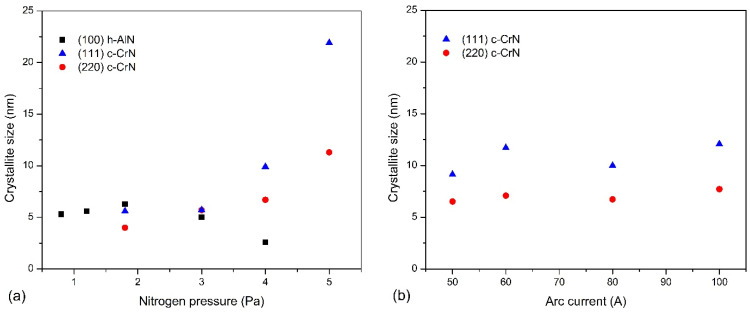
The crystallite size of (100) h-AlN and (111) and (220) c-CrN phases in AlCrN coating synthesized at: (**a**) different nitrogen pressure, (**b**) different arc current.

The crystallite sizes of both phases present in coatings investigated were calculated using the Scherrer equation, as shown in Figure 4. Crystallites in the coatings formed under nitrogen pressure up to 3 Pa are small, up to about 7 nm, Figure 4a. The size of the crystallites determined from the (100) h-AlN plane increases slightly from about 5 nm (p_N2_ = 0.8 Pa) to about 7 nm (p_N2_ = 1.8 Pa), and then decreases to about 3 nm (p_N2_ = 4.0 Pa). Figure 3 shows a significant reduction in the intensity of the diffraction line and its widening for this coating. The crystallites determined in planes (111) and (220) of the c-CrN phase increase from about 4–5 nm (p_N2_ = 1.8 Pa) and reach about 11 nm and 22 nm (p_N2_ = 5.0 Pa), respectively.

The coatings formed at different arc currents under nitrogen pressure of 4 Pa crystallize in the CrN cubic structure, Figure 3b. Therefore, the crystallite sizes for the diffraction lines from planes (111) and (220) were calculated, Figure 4b. Regardless of the arc current used to produce the coatings, the crystallite sizes are similar to each other and amount to about 7–8 nm and 9–12 nm, respectively. The trend of changes in the size of crystallites with the change of the arc current was not recorded.

### 3.2. Surface Morphology

**Figure 5:**
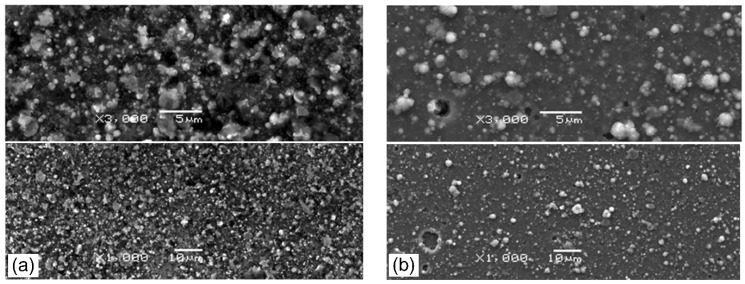
Morphology of of AlCrN coatings synthesized at: (**a**) p_N2_ = 0.8 Pa and (**b**) p_N2_ = 5 Pa. Each picture shows the area recorded at magnification: 1000× (**bottom**) and 3000× (**top**).

**Figure 6:**
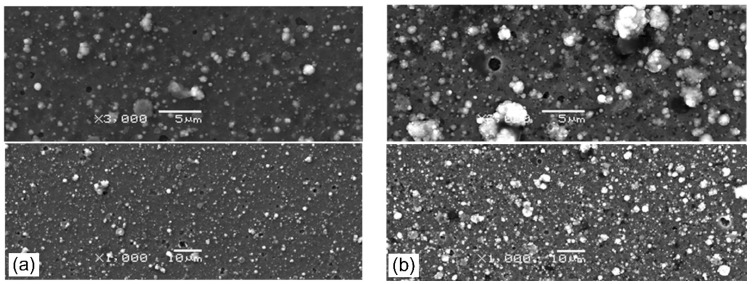
Morphology of of AlCrN coatings synthesized at: (**a**) Ic = 50 A and (**b**) Ic = 100 A. Each picture shows the area recorded at magnification: 1000× (**bottom**) and 3000× (**top**).

The surface morphology of the coatings formed at p_N2_ = 0.8 Pa (Figure 5a), p_N2_ = 5 Pa (Figure 5b) and Ic = 50 A (Figure 6a) and Ic =100 A (Figure 6b) is shown below. Each picture shows the area recorded at magnification: 1000× (bottom) and 3000× (top). A large number of surface defects, macroparticles, and craters were observed on the surface of all coatings. These surface defects are a disadvantage of the commonly used coating deposition method—cathodic arc evaporation. Cathodic arc discharge occurred at cathode spots. In these spots, the material of the cathode material is converted from solid to plasma extremely quickly as a result of ion bombardment and Joule heating, and macroparticles or droplets are emitted [37]. These droplets can group in the plasma and deposit on the surface of the coating [38]. Many macroparticles of different sizes were observed on the coating surface. There are also visible craters in places of removed macroparticles. The macroparticle shapes are diverse: elongated, irregular, but mostly spherical. The sizes of the particles vary greatly from fractions of a micrometer to several micrometers, with the vast majority reaching dimensions up to 1 µm. The macroparticles and their size distribution do not depend on the cathode, but on the method of coating formation, i.e., cathodic arc evaporation. AlTiN coatings have a similar size distribution of macroparticles on the coating surface [38].

The analysis of Figure 5 and Figure 6 shows that the coatings formed with higher nitrogen pressure have fewer surface defects. A similar effect for AlTiN coatings was observed by Cai et al. [38], while Wang et al. [30], who investigated AlCrN coatings, found the opposite effect. 

For coatings synthesized at different arc currents, the opposite effect is visible. On the coating surface formed at the arc current of 50 A, fewer defects are observed, as shown in Figure 6a, than on the coating formed at higher current, shown in Figure 6b.

**Figure 7:**
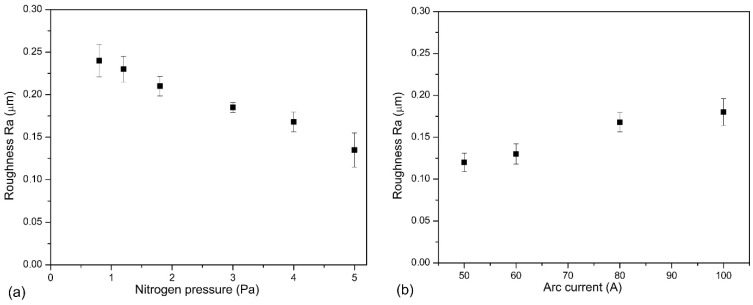
Roughness parameter Ra of AlCrN coatings formed at: (**a**) various pressure of nitrogen, (**b**) various arc current.

The roughness of the coatings depends on the development of its surface, e.g., the amount of surface defects of the coatings, i.e., macroparticles and craters. The analysis of Figure 5a,b shows a certain regularity in the coatings synthesized at higher arc current, where a higher amount of macroparticles is observed. The opposite effect, i.e., a smaller amount of macroparticles, is observed in the second set of coatings—synthesized at higher nitrogen pressure. This change in the surface quality of the coating is reflected in the roughness parameter Ra. There is a noticeable reduction in the Ra parameter, as shown in Figure 7. The initial high Ra value, about 0.24 µm, for the coating synthesized at p_N2_ = 0.8 Pa, reduces almost twice to 0.13 µm (p_N2_ = 5 Pa), as shown in Figure 7a. In the case of coatings deposited at various arc current, the opposite effect is observed, increase Ra value from about 0.12 (Ic = 50 A) to about 0.18 µm (Ic = 100 A). Such a high roughness value is comparable with the results presented by other authors for aluminum-doped chromium nitrides or titanium nitrides [38,39] deposited using the cathodic arc evaporation. CrN coatings formed by the same method at p_N2_ = 1.8 Pa are characterized by lower Ra roughness parameter of 0.08 μm [40]. This means that doping the aluminum of the CrN coating causes more than a 200% increase in surface roughness.

### 3.3. Mechanical Properties

The durability of tools or machine surfaces modified with thin coatings depends on the properties of these coatings. They determine, among other things, the reliability of the devices of load-carrying at the tribological point. The deposited coating generally promotes a favorable compressive stress. In the case of thin coatings, the most frequently used methods of measuring their hardness and modulus of elasticity are the nano-and micro-indentation methods. It should also be remembered that the penetration depth should be selected so that the substrate properties do not affect the results of the coating hardness measurement [41,42,43].

The indentation test allowed to determine hardness (H) and Young’s modulus (E), the basic mechanical properties of the coatings. A clear dependence of these values on the nitrogen pressure in which the coatings are formed was observed, Table 1. The coating deposited at p_N2_ = 0.8 Pa has the lowest hardness, about 17.4 GPa. It increases almost linearly with the nitrogen pressure during their formation, reaching the value of 27.7 GPa at p_N2_ = 5 Pa. Young’s modulus is characterized by a similar tendency and is 205 GPa the coating formed at p_N2_ = 0.8 Pa, and for the coating synthesized in p_N2_—5 Pa it is almost 50% more—304 GPa. The wear resistance can be predicted from the coating hardness, one of the most important mechanical features. The results of many authors’ tests show that the combination of the two parameters H and E enables a much better assessment of the coatings in terms of their wear resistance. The two best known are H/E [44] and H^3^/E^2^ [45] rates. H/E ratio (knows as elastic strain to failure) ratio and shows the elastic behavior of the coating in contact with the load used, while H^3^/E^2^ is known as resistance to the plastic indentation. There are also other indicators, e.g., (H^2^/2E—modulus of resilience or (H/E)^2^—transition on mechanical contact—elastic to plastic) that have some importance for the abrasion resistance of materials [46]. 

The H/E ratio is about 0.085 for coatings formed at pressures not higher than 1.8 Pa. The coatings formed in atmosphere with nitrogen pressure from 3 Pa to 5 Pa are characterized by a higher ratio, ranging from 0.091 to 0.094. In the case of the coating resistance to plastic deformation H^3^/E^2^, an almost linear increase is observed from 0.12 GPa (p_N2_ = 0.8 Pa) to 0.23 GPa (p_N2_ = 5Pa).

The mechanical properties of the coatings, both hardness and Young’s modulus formed at various arc currents show a similar tendency, as shown in Table 2. Above features increase with arc current in deposition process. This is the reason for the nearly identical H/E and H^3^ E^2^ ratios for the coatings. They are in the range from 0.092 to 0.099 and from 0.22 GPa to 0.24 GPa. Due to the uncertainty of measurement, it can be assumed that they are the same.

The trend of changes in hardness of coatings formed under different nitrogen pressures is the same, and the hardness values are slightly lower than those presented in Ref. [30], where it ranges from about 22 GPa to about 33 GPa. The HIPIMS coatings are characterized by a hardness ranging from about 11 GPa for low nitrogen flow rates to about 29 GPa for N_2_/Ar = 140% [25]. Differences in hardness may be related to the method of forming the coatings, but also to the use of a cathode with a different chemical composition—Al_50_Cr_50_. As already mentioned, the coatings formed at different arc currents show a decrease in hardness with increasing arc current [31], which is contrary to our observations. The decrease in hardness is explained by a significant increase in the cathode temperature (arc current up to 150 A), which leads to a larger amount of the droplet phase [31].

**Figure 8:**
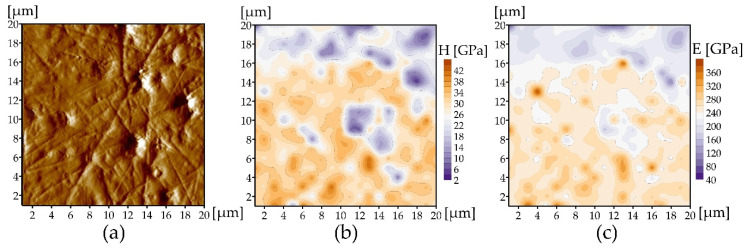
AlCrN coating deposited at p_N2_ = 3 Pa, Ic = 80 A: (**a**) surface morphology after polishing, (**b**) microhardness map, (**c**) Young’s modulus map. Coating area 20 × 20 μm^2^.

The surface of the AlCrN coating formed at p_N2_ = 3 Pa, in terms of roughness, occupies a middle position among the coatings deposited at other nitrogen pressures (Figure 7), but it still contains quite a lot of macroparticles and craters (Figure 6). The surface can be described as completely covered with macroparticles with a diameter of 100 nm–3 µm, among which there are free areas of the surface no more than 1–2 µm. The probability of indentation into a macroparticle is much higher than into a free surface during NI. Therefore, to determine E and H without particles, the surfaces of the coatings were polished. After polishing, the macroparticles disappear and depressions appear on the topography in place of the largest of them, as is always the case with softer phases on the metallographic polished surface (Figure 8a). The fact that large macroparticles can spread throughout the thickness of the coating is shown in [47]. In the topography image, most of the coating is free from particles and much less of the depression from the macroparticles on the remaining surface. E and H maps show the distribution of values corresponding to this surface. Most of the surface is orange (Figure 8b,c), which in these images corresponds to high values (240–360 GPa for E and 26–40 GPa for H). There are areas of lilac color between them, corresponding to the remains of large particles, some of which left inside the coating (Figure 8b,c) (20–160 GPa for E and 2–16 GPa for H). The mean value of E for the polished surface is 241 ± 40 GPa. The mean value of H is 27.5 ± 7.4 GPa. Lower E and H correspond to particles, possibly containing metals Cr and Al (Cr was established by XRD), or traces of the metastable γ-phase Al_2_O_3_, which coincides in peaks with c-CrN and Cr.

The adhesion of a coating to the surface of a substrate (connected with the coating bond strength) determines the difficulty of removing the coating from the substrate. The more difficult it is to remove the coating, the better its adhesion. To improve the adhesion of the coatings, an intermediate layer (metallic adhesive layer) is often used between the substrate and the coating. One of the most common methods of testing the strength of the bond between the coating and the substrate is the scratch test. The critical load Lc_2_ for coatings formed under various nitrogen pressure is from about 77 N to about 98 N (Table 1). A distinct Lc_2_ maximum is observed. An increase in the critical load of Lc_2_ under nitrogen pressure from about 77 N (p_N2_ = 0.8 Pa) to about 98 N (p_N2_ = 3 Pa) can be noticed, and then a decrease to about 80 N. AlCrN and CrN coatings generally present very good adhesion, the critical load Lc_2_ exceeds 80 N [30]. The coatings formed at various arc current are characterized by an almost linear increase in critical load Lc_2_ from about 88 N to about 97 N, Table 2.

**Figure 9:**
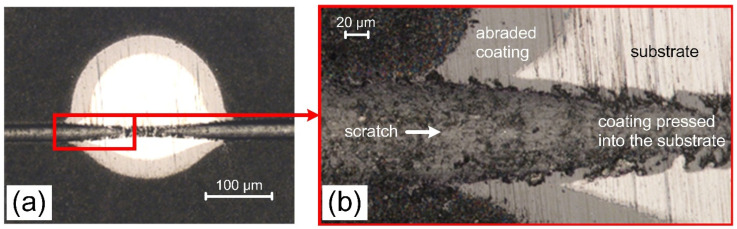
Optical microscope micrographs of the result of the combined test (scratch test and spherical abrasion test) for AlCrN coating deposited at _pN2_ = 0.8 Pa and Ic = 80 A (**a**), and (**b**) the enlarged image of the red rectangle in Figure 9a. Image of the coating from the top.

The spherical abrasion test allows to determine the coating thickness. The combination of the spherical abrasion test and the scratch test proposed by Panjan et al. [48] allows the assessment of both the characteristics of the coating structure and the deformation of the coating and the substrate under proposed load. The above double test was applied to the coating characterized by the lowest critical force Lc_2_ (Table 1) formed at p_N2_ = 0.8 Pa and Ic = 80 A, as shown in Figure 9. Figure 9a shows an image of abrasion by a steel ball in an abrasion test and a scratch in the place where the normal load of the Rockwell indenter was 70 N, which is slightly below Lc_2_. Despite relatively low critical load Lc_2_ this coating shows excellent adhesion both in the scratch area and in the substrate-ball crater area. No detachment was observed at both the ball abrasion—substrate and the ball abrasion—scratch interface, as shown in Figure 9b. There is noticeable deformation of the coating as well as the substrate, especially in the part including the scratch. In the scratch test, a plastic deformation of the substrate occurs as a result of a gradual increase in the normal force, and thus the scratch depth. As a result, the pressed-out substrate material and cracking of the coating occur at the scratch boundaries. The high stress on the interface between the coating and the substrate may result in the coating delamination. This effect did not occur in the coatings tested. Therefore, it can be concluded that these coatings are characterized by high adhesion, and the AlCrN coating–steel substrate systems have high mechanical stability. The coating adheres tightly to the surface, even the one pressed into the bottom of the scratch.

**Figure 10:**
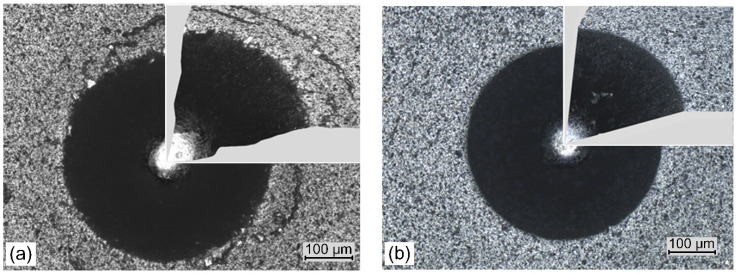
Daimler-Benz adhesion test results, SEM images of Rockwell indents for coatings formed at: (**a**) p_N2_ = 0.8 Pa and (**b**) p_N2_ = 5 Pa.

The standard Daimler–Benz test involves pressing a Rockwell indenter into the coating with a load of approximately 1470 N. Optical analysis of the of cracks and peeling of the coating at the edges of the impression allows these failures (corresponding to coating adhesion) to be assigned to one of the six adhesion patterns. Figure 10 shows images of indentations of the coatings formed at the lowest (Figure 10a) and highest nitrogen pressure, Figure 10b. In the coating deposited at nitrogen pressure of 0.8 Pa few short radial cracks, small areas of coating delamination and circular cracks in the peripheral region of indentation are observed, Figure 10a. Due to it, the coatings are characterized by adhesion consistent with the HF2 damage pattern. In coating formed at the nitrogen pressure of 5 Pa, the above defects are not visible.

**Figure 11:**
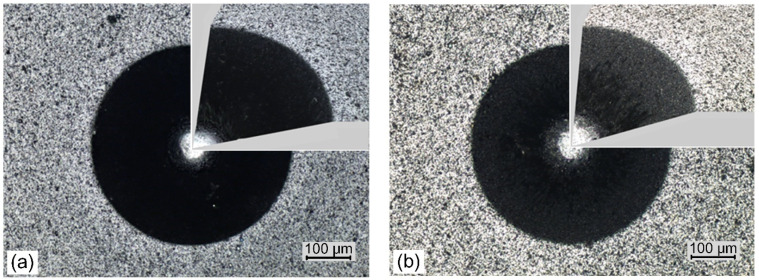
Daimler-Benz adhesion test results, SEM images of Rockwell indents for coatings formed at: (**a**) Ic = 50 A and (**b**) Ic = 100 A.

Only a slight pile-up of material can be observed around the indents, especially in Figure 10a. Adhesion of the coatings is consistent with the HF1 damage pattern. Likewise, the coatings formed at different arc currents have similar properties. Only the coatings deposited at the extreme arc currents of 50 A (Figure 11a) and 100 A (Figure 11b) are shown. These coatings also show an adhesion consistent with the HF1 damage pattern. In all the images it is difficult to even notice the occurrence of the most common coating defects—short radial cracks. This type of damage, or rather the lack of it, qualifies the coatings for industrial applications.

### 3.4. Friction and Wear

The tests carried out on all coatings as a function of the friction distance showed that their coefficient of friction fluctuated without any clear tendency. The changes in the friction coefficient during the test were similar for all coatings. There were two stages of the friction process: the running-in stage (distance approx. 300 m), where there were larger changes and an increase in the friction coefficient to a value of approx. 0.6–0.7, and the steady-state wear stage, where only slight fluctuations of the coefficient were observed. No sudden changes in the friction coefficient were observed, which would suggest a change in the friction environment or coating damage. The coefficient of friction of coatings formed at different nitrogen pressure, determined in the pin-on-disk system against the counter sample—Al_2_O_3_ ball, shows a fine tendency to decrease from 0.68 ± 0.03 (p_N2_ = 0.8 Pa) to 0.63 ± 0 0.02 (p_N2_ = 5 Pa). The coatings formed at different arc currents are characterized by a similar tendency. The coefficient of friction reaches the highest value of 0.70 ± 0.03 for coatings formed at arc current of 50 A and the lowest value of about 0.64 ± 0.03 for coatings formed at arc current of 100 A. It means that, in both cases, the coefficient of friction only slightly depends on deposition parameters, i.e., nitrogen pressure and arc current.

**Figure 12:**
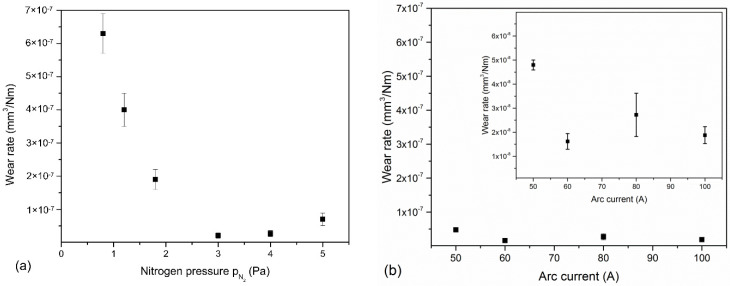
Wear rate of AlCrN coatings formed at: (**a**) various nitrogen pressure, (**b**) various arc current.

The coatings formed at low nitrogen pressure, i.e., 0.8 Pa and 1.2 Pa show relatively high wear rates, about 6.4 × 10^−7^ mm^3^/Nm and 4.0 × 10^−7^ mm^3^/Nm, respectively, Figure 12a. One can observe that wear rate of the coatings decreases, and the coating deposited at 3 Pa shows the lowest wear rate 2.1 × 10^−8^ mm^3^/Nm. Coatings formed at higher nitrogen pressure as well as coatings deposited at various arc currents are characterized by similar values of wear rate ranging from 1.7 × 10^−^^7^ mm^3^/Nm to 4.7 × 10^−^^7^ mm^3^/Nm with a small reducing trend with arc current (Figure 12b). The wear rate of the coatings investigated is low, except for those formed under a low nitrogen pressure. The value of the wear rate, from about 2 × 10^−^^7^ mm^3^/Nm to about 2 × 10^−^^8^ mm^3^/Nm is very low and may suggest possible industrial application of the coatings. For comparison, Reiter et al. [20], examining the coatings formed with the same method, determined wear rates from 2 × 10^−^^6^ mm^3^/Nm (71at% of Al in AlCrN coating) to 8 × 10^−^^6^ mm^3^/Nm (CrN). Lower values of the wear rate, about 3 × 10^−^^7^ mm^3^/Nm, regardless of the applied substrate bias voltage during the formation of CrAlN coatings are presented in Ref. [24]. Antonov, examining the tribological properties of Al_60_Cr_40_N coatings in relation to various counter-samples as well as various test parameters (normal load, sliding speed, measurement temperature), stated that for the Al_2_O_3_ counter-sample, the wear rate was from 3 × 10^−6^ mm^3^/Nm to 2 × 10^−7^ mm^3^/Nm depending on sliding speed.

## 4. Discussion

### 4.1. Effect of Nitrogen Pressure

The coating thickness should be greater with increasing nitrogen pressure. Such an increase to a pressure of 3 Pa is observed, and then the thickness of the coating decreases, Figure 1a. During the movement of ions and particles from the cathode to the substrate, collisions with atoms of reactive gas, i.e., nitrogen, occur. At higher pressures, the probability of collisions is higher, which reduces the mobility and energy of particles, so the free path is shorter and the time to reach the substrate is longer [14]. This reduces the deposition rate of the coatings, i.e., coating thickness. Similar results for the coatings were presented previously for the AlCrN [24,30] and TiAlN [38] coatings. With the nitrogen pressure increase, the chemical composition of the deposited coating also changes, Figure 2a. The nitrogen concentration in the coating increases, reaching the value of about 46 at.%, and the concentration of aluminum and chromium decreases, as well as Al/(Al + Cr) rate. This means that there is loss of aluminum in the coating. The lower atomic mass of aluminum in comparison to chromium in the case of collisions with nitrogen ions results in greater scattering of aluminum ions, and thus less of its condensation on the substrate, as shown earlier [49].

In the coatings formed at p_N2_ not higher than 4 Pa, the presence of the hexagonal AlN phase was found. At low pressures, this is due to a nitrogen deficiency during coating formation. With the increase of nitrogen pressure, the intensity of the h-AlN and h-Cr_2_N diffraction lines decreases and is small in the coatings formed at p_N2_ = 3 and 4 Pa. At the same time, an increase in the intensity of diffraction lines originating from cubic CrN is observed. The transformation of the B1 → B4 structure takes place when the AlN content in the AlCrN coatings is from 60% to 77% [20,22,23]. The tested coatings were formed from the Al_70_Cr_30_ cathode, and their Al/(Al + Cr) rate in the coating was only slightly different from that in the cathode. It means that, in the above conditions, the formation of h-AlN phase or mixture of h-AlN and c-AlN is authorized.

The phase composition of the coating is determined not only by the aluminum concentration. It has been found that the hexagonal phase may also arise as a result of other factors, such as vacancies or compressive stresses [50], which usually occur in PVD coatings, as well as the size of crystallites. It was found shown that the formation of the hexagonal phase is preferred in the case of coatings characterized by fine crystallites [51]. In TiAlN coating with aluminum content in the range 0.55 to 0.70, crystallites with dimensions of 3–5 nm favor the formation of the hexagonal AlN phase, while crystallites with dimensions above 8 nm favor the formation of the cubic phase [51]. Thus, it is the crystallite size (Figure 4a) that may determine the hexagonal phase formation during coating growth.

This phase adversely affects the mechanical properties of the coating. The coatings with c-AlN phase are characterized by higher hardness than those with h-AlN phase. Presumably, it is also influenced by density, and the hexagonal phase, compared to the cubic one, is about 18% lower [20].

The increase in hardness can be related to the increase in crystallinity of the c-CrN phase, visualized by the intensity of diffraction lines. The H/E ratio of coatings formed at pressures of 0.8–1.8 Pa is about 0.085, while for p_N2_ ≥ 3 Pa it increases to 0.094. In the H/E ratio, two areas can be distinguished: elastic (H/E > 0.1) and plastic (H/E < 0.1) and [52]. Better wear resistance is predicted for higher H/E and H^3^/E^2^ ratios. This suggests that coatings deposited at higher nitrogen pressure should be more wear resistant. Indeed, the wear rates of coatings formed at p_N2_ ≥ 3 Pa are significantly lower than other coatings, Figure 12.

The surface roughness of rubbing elements affects the tribological properties of the coatings. Greater roughness intensifies friction processes, accelerating the wear process. There is a clear correlation between the wear results of the coatings (Figure 12) and their roughness, Figure 7a. Another factor that determines wear resistance is hardness. The greater the hardness, the greater the wear resistance is. Although the coating is heterogeneous in terms of mechanical properties (Figure 8) and the hardness ranges from 26 GPa to 40 GPa, good wear resistance of the coatings should be associated with their hardness. This large hardness dispersion is related to surface defects (Figure 5) characteristic of the coating deposition method.

Two methods of assessing the adhesion of the coating, the scratch test enabling numerical determination of adhesion as critical load (Table 1) and the Daimler–Benz test (Figure 10), where the assessment is based on microscopic analysis of the damage to the coating around the Indentation, showed the same result—all coatings are characterized by very good properties. The Daimler–Benz test uses a high normal load of approximately 1470 N. Plastic deformation of the coating and the substrate results from the transformation of the kinetic energy of the moving indenter. This leads to pile up around the indenter which can generate high tensile stresses around the indentation, as in Figure 10a. As a result of these stresses, apart from coating delamination, circular cracks of the coating may occur [53]. This effect may be a consequence of both the high roughness of the coating and its low hardness. On the contrary, the coating formed at p_N2_ does not show similar damage.

### 4.2. Effect of Arc Current

Thickness of the coatings increases with arc current, Figure 1b. This is due to the greater energy of the arc spots and the plasma density, which is a result of the higher arc current used, as reported by Lan et al. [31] investigating AlCrN coatings obtained by the ion arc method. In the case of the coatings synthesized with a higher arc current, the surface is more developed. More surface defects can be observed there, Figure 6b. Higher target energy and temperature, which helps to increase the emission of the droplets from cathode material is connected with higher arc current. Figure 7b confirms this unfavorable arc current effect on the surface quality of the coating. 

The coatings formed at different currents are characterized by the cubic structure of B1 of the CrN phase, Figure 4b. This is consistent with reports that the solubility of Al in solid fcc-CrN is 60–77% [20,21], so the Al atom occupies the Cr sites in the CrN lattice, forming a substitution solid solution, leading to solid solution strengthening. Despite using an arc current of 50 A to 100 A, the diffraction patterns remain similar. The mutual ratio of the intensities of the diffraction lines is similar. This suggests that an increase in the arc current does not significantly modify the crystal lattice. The analysis of the position of the (111) and (200) diffraction lines shows only a slight shift towards higher angles (Figure 4b), which suggests no change in the stress state in the coating. The full width at half maximum intensity of the line seems similar, so an increase in the arc current does not change the crystallite size, Figure 5b. This effect is the opposite to that described in Ref [31], although the determined crystallite sizes are similar. The increase in the arc current did not increase the plasma energy allowing for grain size differentiation and internal stresses in the coating.

As mentioned earlier, plasma with higher energy and density that can alter the properties of the coating is associated with a higher arc current. Under such conditions, it is possible to increase the density of the coating, also by reducing the size of the crystallites. As a result, this should result in coating hardness increase. Indeed, the hardness of the coatings was greater for higher the current value. However, the increase, although obvious, was within the range of the hardness measurement uncertainty, Table 2. This may be due to the fact that the crystallite sizes were comparable for all coatings (Figure 4b), so it would be difficult to connect a change in hardness with the Hall–Petch relationship. Probably one should take into account another effect related to the higher energy of the incident ions, the increase in the density of the coatings. Lan et al. [31] formed coatings with arc currents from 100 A to 150 A and recorded the opposite effect, reducing the hardness with increasing current during coating formation. They found that at such a high arc current, increased droplet ejection from the cathode and deterioration of the surface of the coating quality occurs. It seems that the reduction of hardness by defects on the surface and in the whole coating by macroparticles is stronger than the improvement connected with plasma energy and density. As mentioned, the coatings described in this study were formed at arc currents from 50 A to 100 A. This probably resulted in the observation of opposite changes in hardness. The amount of macroparticles on the surface increases significantly, which was also observed in Figure 3b. The macroparticles on the surface are also accompanied by similar defects in the volume of the coating as well as by voids [46]. Sometimes, larger macroparticles extend through the entire thickness of the coating, deteriorating the mechanical and electrochemical properties.

Both H^3^/E^2^ rate and H/E rate as a predictor of the wear resistance of the coatings have similar values, of about 0.23 and about 0.1 (Table 2), regardless of the arc current during formation, respectively. The values obtained for the tested coatings indicate that they should have good wear resistance, comparable for all coatings. Indeed, the wear resistance shown in Figure 12b is high, the wear rate about (2 to 4) × 10^8^ mm^3^/Nm close to each other.

The adhesion of the coatings (Table 2) shows an increasing trend from about 88 N (Ic = 50 A) to about 97 N (Ic = 100 A). This means that the adhesion is very good. The increase in adhesion may be related to many factors: an increase in the coating density and its hardness as well as higher energy of incident particles. The higher energy of the particles can even result in their shallow implantation into the substrate, which creates a well-adherent adhesive layer. The implantation depth is usually 5–15 nm [53], although it can be as high as 50 nm. It depends on the energy of the ions. With higher energy, the stresses in the coating can be increased, which can lead to reduced adhesion. 

The results of Daimler–Benz test confirm the very good adhesion of investigated coatings. Regardless of the arc current value during their formation, the damage image showed the highest class of HF1 adhesion, Figure 11. It is possible that one of the reasons for this was the use of a thin gradient Cr-N adhesive layer with an increasing nitrogen concentration in this layer. A similar result was reported in [54] where pure chromium was used.

The above technological works of forming coatings at various nitrogen pressures and arc currents with a wide range of their values, as well as examining their structure, mechanical and tribological properties, were to indicate a set of technological parameters enabling the formation of the coatings with the best properties. The coatings were applied to HS6-5-2 high speed steel substrates (EU EN and DIN, M2—USA), which is widely used for tools. Comparing the properties of the coatings obtained in this way with those of commercially used coated tools may contribute to their incorporation into the set of coatings used in practice.

## 5. Conclusions

In this work, we investigated two sets of coatings: deposited at different nitrogen pressure (constant arc current) and different arc currents (constant nitrogen pressures). The deposition method was cathodic arc evaporation. The effect of these factors on structure features, i.e., phase composition and crystallite size, as well as coating microstructure, mechanical, and tribological properties, was studied. The results indicate that with increasing nitrogen pressure during coating formation the h-AlN phase gradually disappears. Changing the arc current does not affect the type of phase formed. The coatings formed under both increasing nitrogen pressure and arc current are characterized by increasing hardness. They show very good adhesion, the critical force from the scratch test is above 77 N, and according to the Daimler–Benz test, most coatings show HF1 class. The coatings formed at higher pressure exhibit good wear resistance.

The effect of nitrogen pressure during AlCrN coating deposition from AlCr cathodes of different chemical composition is known. The further direction of technological and research works is the doping of such coatings with other metallic and non-metallic elements, creating four-and more-component coatings with excellent mechanical, tribological, and electro-chemical properties for application on tools for dry machining.

## Figures and Tables

**Table 1:** Mechanical properties of AlCrN coatings formed at various pressure of nitrogen.

Parameter	AlCrN(0.8)	AlCrN(1.2)	AlCrN(1.8)	AlCrN(3.0)	AlCrN(4.0)	AlCrN(5.0)
Hardness H (GPa)	17.4 ± 1.3	18.0 ± 2.3	18.7 ± 2.9	21.8 ± 2.1	24.6 ± 2.2	27.7 ± 2.6
Young’s modulus, E (GPa)	205 ± 13	212 ± 15	218 ± 16	239 ± 14	261 ± 9	304 ± 16
H/E	0.085 ± 0.011	0.085 ± 0.017	0.086 ± 0.019	0.091 ± 0.014	0.094 ± 0.014	0.091 ± 0.013
H^3^/E^2^ (GPa)	0.12 ± 0.04	0.13 ± 0.07	0.15 ± 0.08	0.18 ± 0.07	0.22 ± 0.07	0.23 ± 0.09
Lc_2_ (N)	77.2 ± 1.8	85.0 ± 2.3	84.5 ± 3.5	98.0 ± 3.8	91.0 ± 2.1	79.6 ± 1.8

**Table 2:** Mechanical properties of f AlCrN coatings formed at various arc current.

Parameter	AlCr(50)N	AlCr(60)N	AlCr(80)N	AlCr(100)N
Hardness, H (GPa)	23.3 ± 1.7	24.9 ± 1.9	24.6 ± 2.2	25.4 ± 1.3
Young’s modulus, E (GPa)	235 ± 9	252 ± 10	261 ± 9	275 ± 8
H/E	0.099 ± 0.011	0.099 ± 0.011	0.094 ± 0.014	0.092 ± 0.007
H^3^/E^2^ (GPa)	0.23 ± 0.07	0.24 ± 0.07	0.22 ± 0.07	0.22 ± 0.05
Lc_2_ (N)	88.1 ± 3.3	89.4 ± 1.0	91.0 ± 2.1	97.2 ± 0.9

## Data Availability

The data presented in this study are available on request from the corresponding author.
